# Inverse folding of RNA pseudoknot structures

**DOI:** 10.1186/1748-7188-5-27

**Published:** 2010-06-23

**Authors:** James ZM Gao, Linda YM Li, Christian M Reidys

**Affiliations:** 1 Center for Combinatorics, LPMC-TJKLC, Nankai University, Tianjin 300071, China

## Abstract

**Background:**

RNA exhibits a variety of structural configurations. Here we consider a structure to be tantamount to the noncrossing Watson-Crick and **G-U**-base pairings (secondary structure) and additional cross-serial base pairs. These interactions are called pseudoknots and are observed across the whole spectrum of RNA functionalities. In the context of studying natural RNA structures, searching for new ribozymes and designing artificial RNA, it is of interest to find RNA sequences folding into a specific structure and to analyze their induced neutral networks. Since the established inverse folding algorithms, RNAinverse, RNA-SSD as well as INFO-RNA are limited to RNA secondary structures, we present in this paper the inverse folding algorithm Inv which can deal with 3-noncrossing, canonical pseudoknot structures.

**Results:**

In this paper we present the inverse folding algorithm Inv. We give a detailed analysis of Inv, including pseudocodes. We show that Inv allows to design in particular 3-noncrossing nonplanar RNA pseudoknot 3-noncrossing RNA structures-a class which is difficult to construct via dynamic programming routines. Inv is freely available at http://www.combinatorics.cn/cbpc/inv.html.

**Conclusions:**

The algorithm Inv extends inverse folding capabilities to RNA pseudoknot structures. In comparison with RNAinverse it uses new ideas, for instance by considering sets of competing structures. As a result, Inv is not only able to find novel sequences even for RNA secondary structures, it does so in the context of competing structures that potentially exhibit cross-serial interactions.

## 1 Introduction

**Figure 1:**
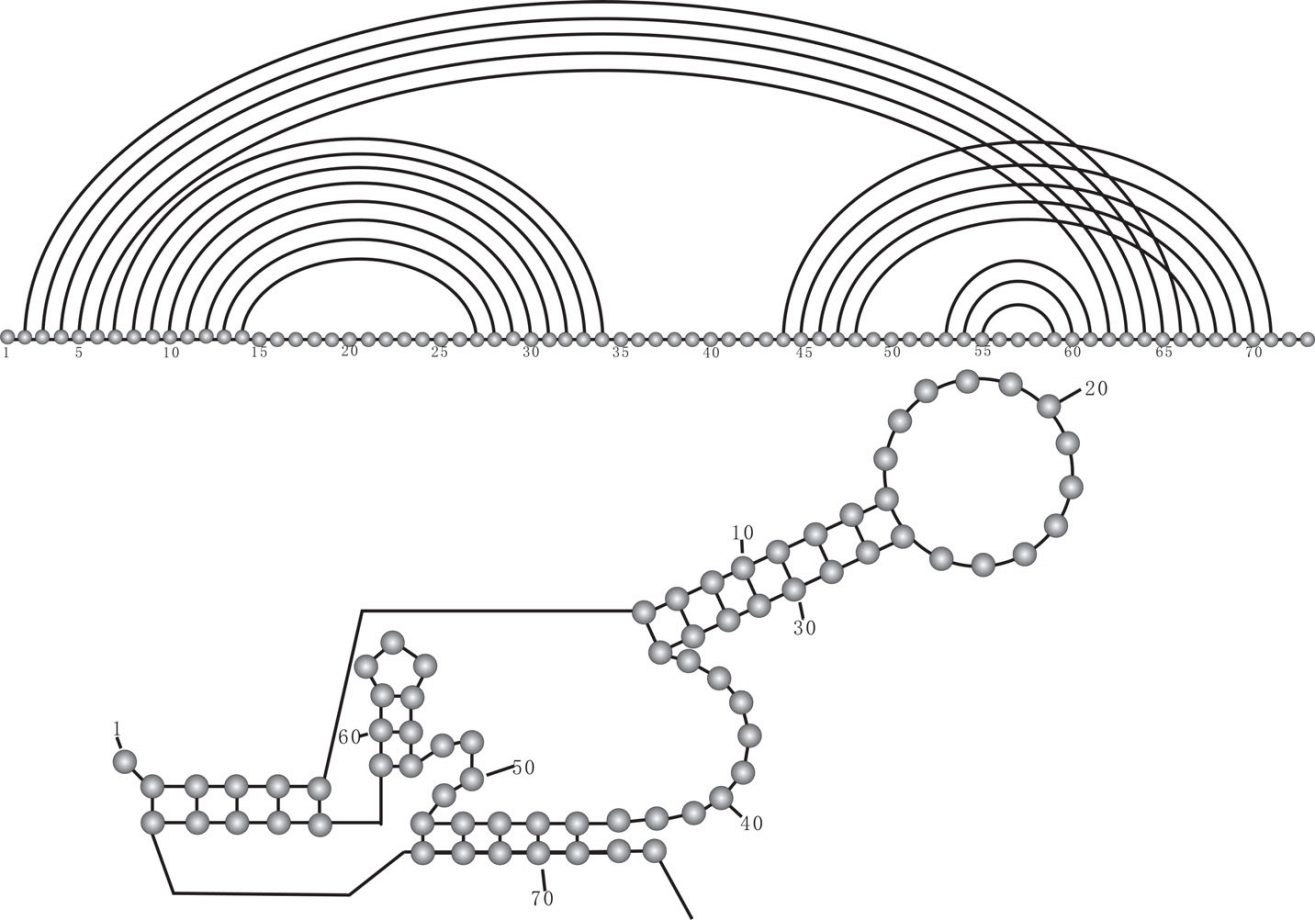
**Representations of RNA structures**. The pseudoknot structure of the glmS ribozyme pseudoknot P1.1 [40] as a diagram (top) and as a planar graph (bottom).

Pseudoknots are structural elements of central importance in RNA structures [1], see Figure 1. They represent cross-serial base pairing interactions between RNA nucleotides that are functionally important in tRNAs, RNaseP [2], telomerase RNA [3], and ribosomal RNAs [4]. Pseudoknot structures are being observed in the mimicry of tRNA structures in plant virus RNAs as well as the binding to the HIV-1 reverse transcriptase in *in vitro *selection experiments [5]. Furthermore basic mechanisms, like ribosomal frame shifting, involve pseudoknots [6].

Despite them playing a key role in a variety of contexts, pseudoknots are excluded from large-scale computational studies. Although the problem has attracted considerable attention in the last decade, pseudoknots are considered a somewhat "exotic" structural concept. For all we know [7], the *ab initio *prediction of general RNA pseudoknot structures is NP-complete and algorithmic difficulties of pseudoknot folding are confounded by the fact that the thermodynamics of pseudoknots is far from being well understood.

**Figure 2:**
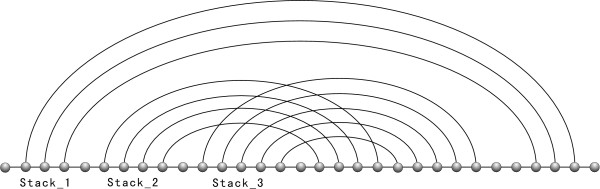
***σ*-canonical RNA structure**. Each stack of "parallel" arcs has to have minimum size *σ*. Here we display a 3-canonical structure.

As for the folding of RNA secondary structures, Waterman *et al *[8,9], Zuker *et al *[10] and Nussinov [11] established the dynamic programming (DP) folding routines. The first mfe-folding algorithm for RNA secondary structures, however, dates back to the 60's [12-14]. For restricted classes of pseudoknots, several algorithms have been designed: Rivas and Eddy [15], Dirks and Pierce [16], Reeder and Giegerich [17] and Ren *et al *[18]. Recently, a novel *ab initio *folding algorithm Cross has been introduced [19]. Cross generates minimum free energy (mfe), 3-noncrossing, 3-canonical RNA structures, i.e. structures that do not contain three or more mutually crossing arcs and in which each stack, i.e. sequence of parallel arcs, see eq. (1), has size greater or equal than three. In particular, in a 3-canonical structure there are no isolated arcs, see Figure 2.

The notion of mfe-structure is based on a specific concept of pseudoknot loops and respective loop-based energy parameters. This thermodynamic model was conceived by Tinoco and refined by Freier, Turner, Ninio, and others [13,20-24].

### 1.1 *k*-noncrossing, *σ*-canonical RNA pseudoknot structures

**Figure 3:**
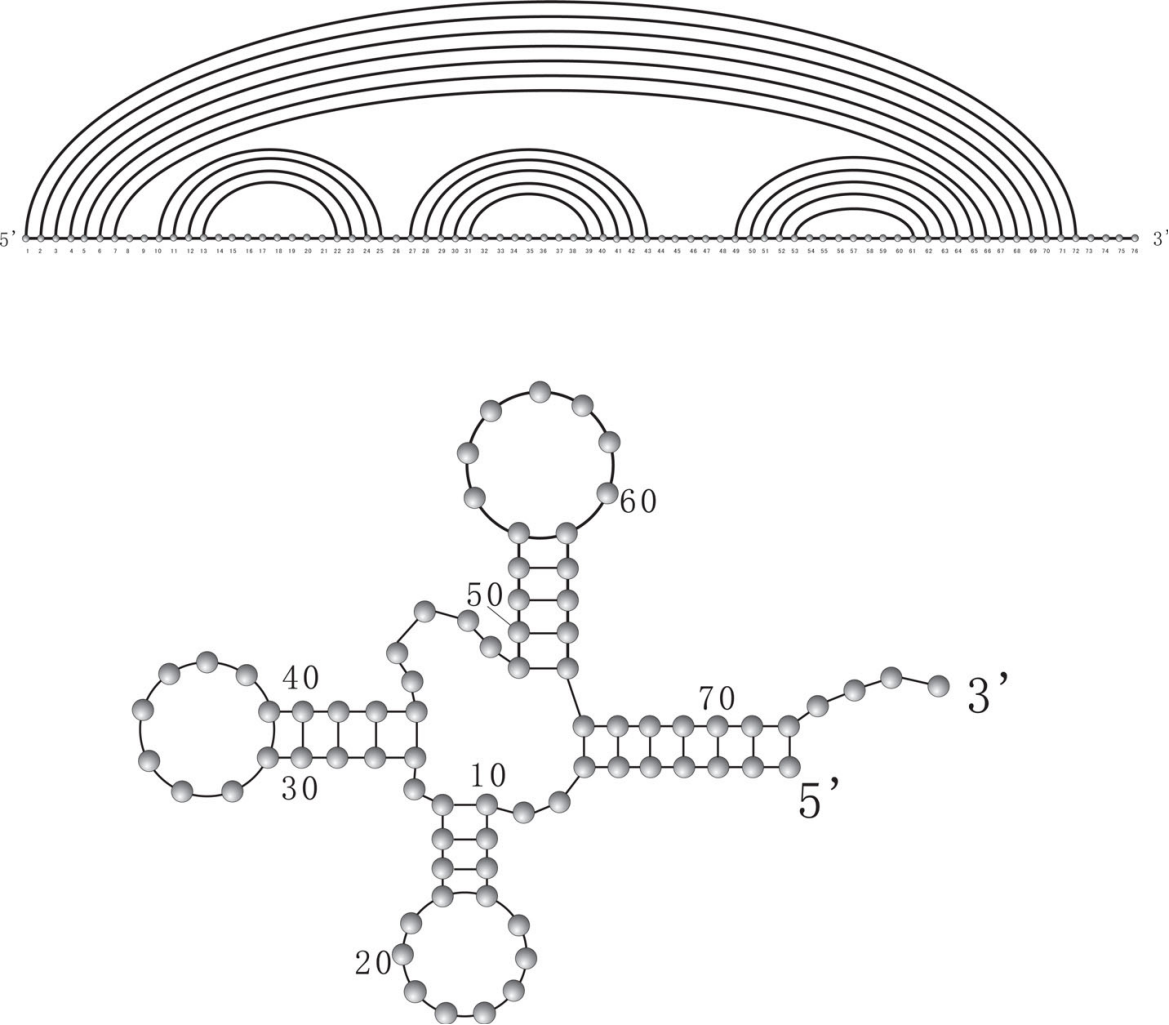
**The phenylalanine tRNA structure**. The phenylalanine tRNA secondary structure represented as 2-noncrossing diagram (top) and as planar graph (bottom).

Let us turn back the clock: three decades ago Waterman *et al. *[25], Nussinov *et al. *[11] and Kleitman *et al. *in [26] analyzed RNA secondary structures. Secondary structures are coarse grained RNA contact structures, see Figure 3.

**Figure 4:**
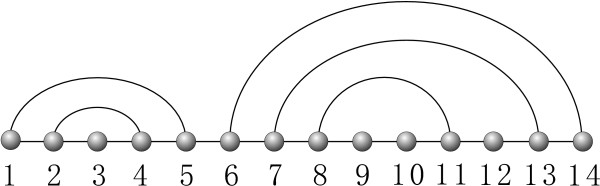
**Secondary structure**. Secondary structures are particular *k*-noncrossing diagrams, 2-noncrossing diagrams exhibit no crossings at all, therefore RNA secondary structures coincide with 2-noncrossing diagrams having minimum arc-length two.

RNA secondary structures as well as RNA pseudoknot structures can be represented as diagrams, i.e. labeled graphs over the vertex set [*n*] = {1, ..., *n*} with vertex degrees ≤ 1, represented by drawing its vertices on a horizontal line and its arcs (*i, j*) (*i < j*), in the upper half-plane, see Figure 4 and Figure 1. Given an arc (*i, j*) we refer to (*j - i*) as its arc-length.

Here, vertices and arcs correspond to the nucleotides **A**, **G**, **U**, **C **and Watson-Crick (**A-U**, **G-C**) and (**U-G**) base pairs, respectively.

**Figure 5:**
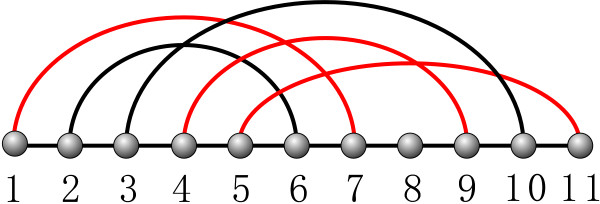
***k*-noncrossing diagrams**. We display a 4-noncrossing diagram containing the three mutually crossing arcs (1, 7), (4, 9), (5, 11) (drawn in red).

In a diagram, two arcs (*i*_1_, *j*_1_) and (*i*_2_, *j*_2_) are called crossing if *i*_1 _<*i*_2 _<*j*_1 _<*j*_2 _holds. Accordingly, a *k*-crossing is a sequence of arcs (*i*_1_, *j*_1_), ..., (*i*_*k*_, *j*_*k*_) such that *i*_1 _<*i*_2 _< ... <*i*_k _<*j*_1 _<*j*_2 _< ... <*j*_*k*_. We call diagrams containing at most (*k *- 1)-crossings, *k*-noncrossing diagrams, see Figure 5.

RNA secondary structures exhibit no crossings in their diagram representation, see Figure 3 and Figure 4, and are therefore 2-noncrossing diagrams satisfying some minimum arc-length condition. An RNA pseudoknot structure is therefore a *k*-noncrossing diagram for some *k *satisfying some minimum arc-length condition.

A structure in which any stack has at least size *σ *is called *σ*-canonical, where a stack of size *σ *is a sequence of "parallel" arcs of the form(1)

**Figure 6:**
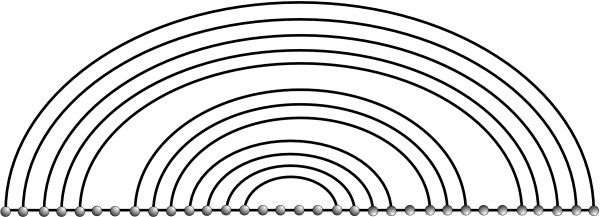
**Stems**. A stem composed by a sequence of three nested stacks. Note that respective stacks only have to be separated by isolated nucleotides on either the left hand side or the right hand side but not necessarily both.

A sequence of consecutive stacks, separated by unpaired nucleotides,  i.e. where

is called a stem of length *r*, see Figure 6.

As a natural generalization of RNA secondary structures *k*-noncrossing RNA structures [27-29] were introduced. A *k*-noncrossing RNA structure of length *n *is *k*-noncrossing diagram over [*n*] without arcs of the form (*i, i *+ 1). In the following we assume *k *= 3, i.e. in the diagram representation there are at most two mutually crossing arcs, a minimum arc-length of four and a minimum stack-size of three base pairs. The notion *k*-noncrossing stipulates that the complexity of a pseudoknot is related to the maximal number of mutually crossing bonds. Indeed, most natural RNA pseudoknots are 3-noncrossing [30].

### 1.2 Neutral networks

**Figure 7:**
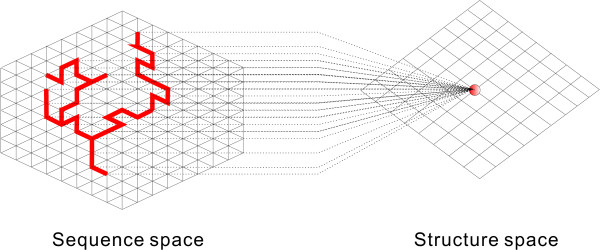
**Neutral network in sequence space**. We display sequence space (left) and structure space (right) as grids. We depict a set of sequences that all fold into a particular structure. Any two of these sequences are connected by a red path. The neutral network of this fixed structure consists of all sequences folding into it and is typically a connected subgraph of sequence space.

Before considering an inverse folding algorithm into specific RNA structures one has to have at least some rationale as to why there exists *one *sequence realizing a given target as mfe-configuration. In fact this is, on the level of entire folding maps, guaranteed by the combinatorics of the target structures alone. It has been shown in [31], that the numbers of 3-noncrossing RNA pseudoknot structures, satisfying the biophysical constraints grows asymptotically as *c*_3_*n*^-5^2.03^*n*^, where *c*_3 _*>*0 is some explicitly known constant. In view of the central limit theorems of [32], this fact implies the existence of extended (exponentially large) sets of sequences that all fold into one 3-noncrossing RNA pseudoknot structure, *S*. In other words, the combinatorics of 3-noncrossing RNA structures alone implies that there are many sequences mapping (folding) into a single structure. The set of all such sequences is called the neutral network of the structure *S *[33,34], see Figure 7. The term "neutral network" as opposed to "neutral set" stems from giant component results of random induced subgraphs of *n*-cubes. That is, neutral networks are typically connected in sequence space.

**Figure 8:**
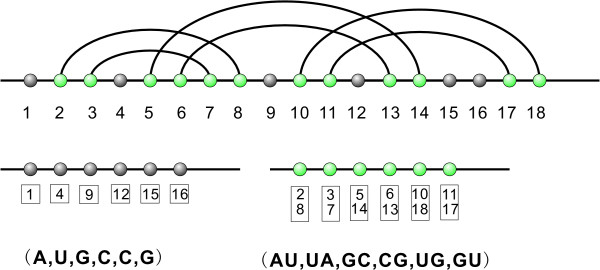
**A structure and a particular compatible sequence**. A structure and a particular compatible sequence organized in the segments of unpaired and paired bases.

By construction, all the sequences contained in such a neutral network are all compatible with *S*. That is, at any two positions paired in *S*, we find two bases capable of forming a bond (**A-U**, **U-A**, **G-C**, **C-G**, **G-U **and **U-G**), see Figure 8. Let *s*' be a sequence derived via a point-mutation of *s*. If *s*' is again compatible with *S*, we call this mutation "compatible".

Let *C*[*S*] denote the set of *S*-compatible sequences. The structure *S *motivates to consider a new adjacency relation within *C*[*S*]. Indeed, we may reorganize a sequence (*s*_1_*, ..., s_n_*) into the pair(2)

where the *u*_*h *_denotes the unpaired nucleotides and the *p*_*h *_= (*s*_*i*_, *s*_*j*_) denotes base pairs, respectively, see Figure 8. We can then view  and  as elements of the formal cubes  and  implying the new adjacency relation for elements of *C*[*S*].

**Figure 9:**
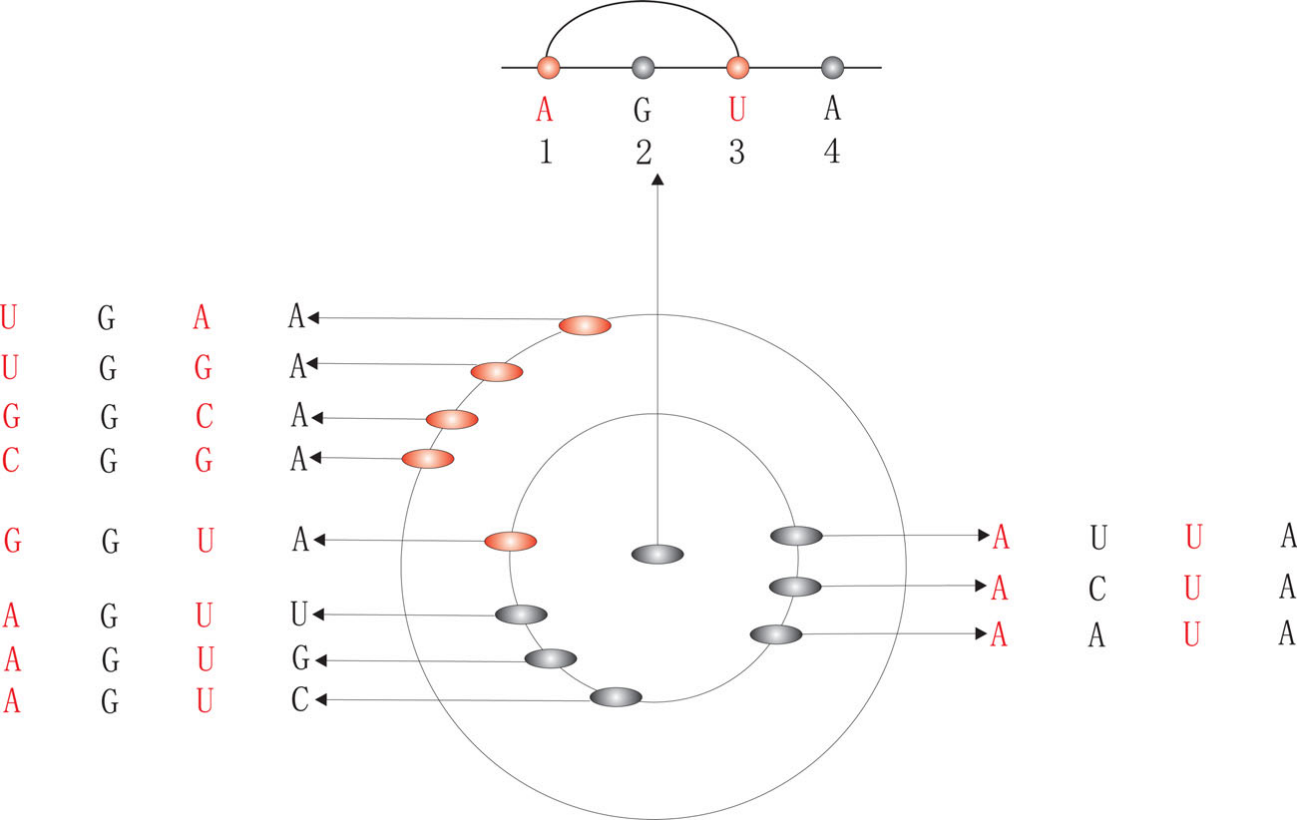
**Diagram representation of an RNA structure and its compatible neighbors**. Diagram representation of an RNA structure (top) and its induced compatible neighbors in sequence space (bottom). Here the neighbors on the inner circle have Hamming distance one while those on the outer circle have Hamming distance two. Note that each base pair gives rise to five compatible neighbors (red) exactly one of which being in Hamming distance one.

Accordingly, there are two types of compatible neighbors in the sequence space u- and p-neighbors: a u-neighbor has Hamming distance one and differs exactly by a point mutation at an unpaired position. Analogously a p-neighbor differs by a compensatory base pair-mutation, see Figure 9.

Note, however, that a p-neighbor has either Hamming distance one (**G-C **↦ **G-U**) or Hamming distance two (**G-C **↦ **C-G**). We call a u- or a p-neighbor, *y*, a compatible neighbor. In light of the adjacency notion for the set of compatible sequences we call the set of all sequences folding into *S *the neutral network of *S*. By construction, the neutral network of *S *is contained in *C*[*S*]. If *y *is contained in the neutral network we refer to *y *as a neutral neighbor. This gives rise to consider the compatible and neutral distance of the two sequences, denoted by *C*(*s*, *s*') and *N*(*s*, *s*'). These are the minimum length of a *C*[*S*]-path and path in the neutral network between *s *and *s*', respectively. Note that since each neutral path is in particular a compatible path, the compatible distance is always smaller or equal than the neutral distance.

In this paper we study the inverse folding problem for RNA pseudoknot structures: for a given 3-noncrossing target structure *S*, we search for sequences from *C*[*S*], that have *S *as mfe configuration.

## 2 Background

For RNA secondary structures, there are three different strategies for inverse folding, RNAinverse, RNA-SSD and INFO-RNA[35-37].

**Figure 10:**
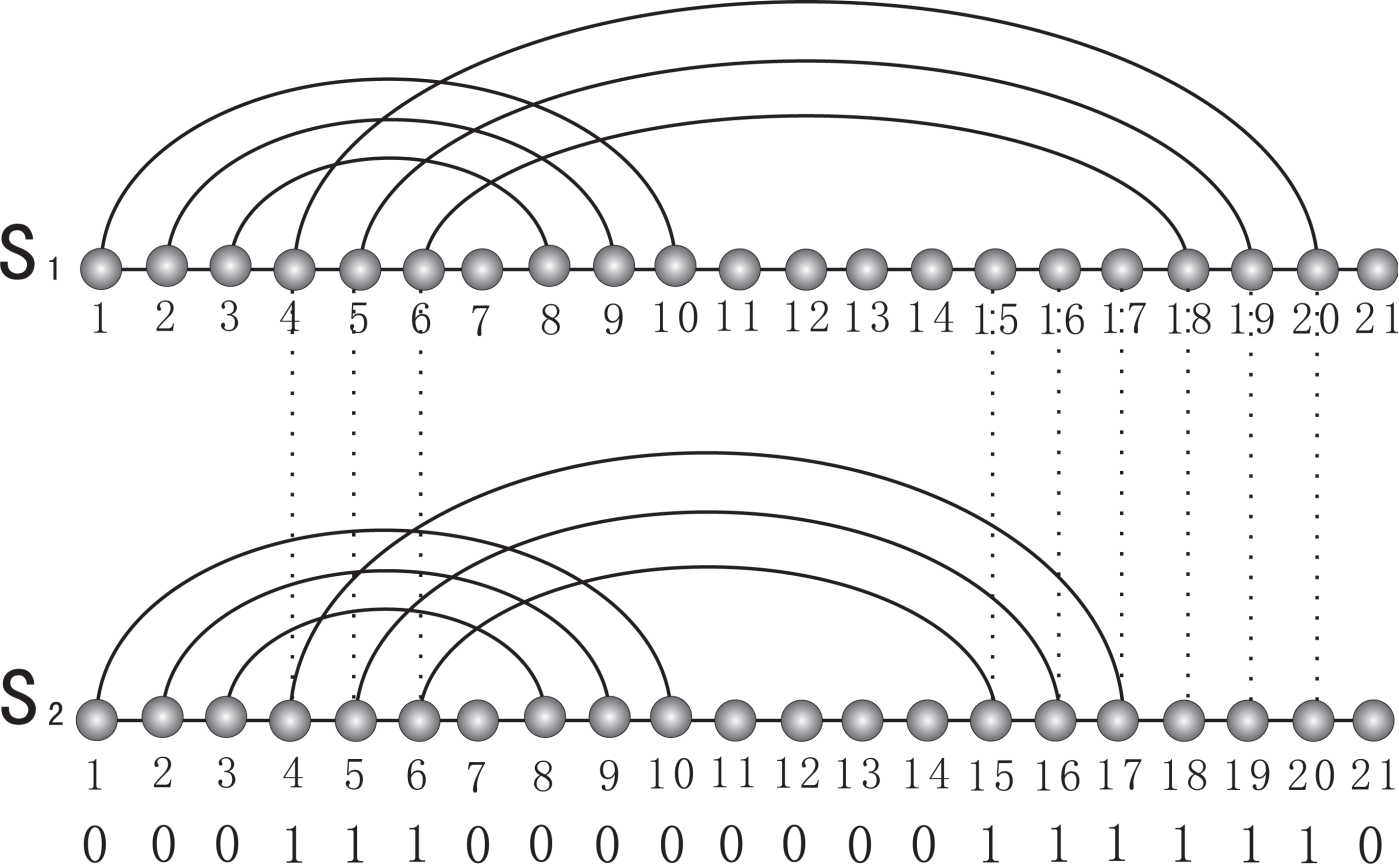
**Distance of two structures**. Positions paired differently in *S*_1 _and *S*_2 _are assigned a "1". There are two types of positions: I. *p *is contained in different arcs, see position 4, (4, 20) ∈ *S*_1 _and (4, 17) ∈ *S*_2_. **II**. *p *is unpaired in one structure and *p *is paired in the other, such as position 18.

They all generate via a local search routine iteratively sequences, whose structures have smaller and smaller distances to a given target. Here the distance between two structures is obtained by aligning them as diagrams and counting "0", if a given position is either unpaired or incident to an arc contained in both structures and "1", otherwise, see Figure 10.

One common assumption in these inverse folding algorithms is, that the energies of specific substructures contribute additively to the energy of the entire structure. Let us proceed by analyzing the algorithms.

**RNAinverse **is the first inverse-folding algorithm that derives sequences that realize given RNA secondary structures as mfe-configuration. In its initialization step, a random compatible sequence *s *for the target *T *is generated. Then RNAinverse proceeds by updating the sequence *s *to *s*', *s*'' ... step by step, minimizing the structure distance between the mfe structure of *s*' and the target structure *T*. Based on the observation, that the energy of a substructure contributes additively to the mfe of the molecule, RNAinverse optimizes "small" substructures first, eventually extending these to the entire structure. While optimizing substructures, RNAinverse does an adaptive walk in order to decrease the structure distance. In fact, this walk is based entirely on random compatible mutations.

**RNA-SSD **inverse folds RNA secondary structures by initializing sequences using three specific subroutines. In the first a particular compatible sequence is generated, where non-complementary nucleotides to bases adjacent to helical regions are assigned. In the second nucleotides located in unpaired positions as well as helical regions are assigned at random, using specific (non-uniform) probabilities. The third routine constitutes a mechanism for minimizing the occurrence of undesired but favourable interactions between specific sequence segments. Following these subroutines, RNA-SSD derives a hierarchical decomposition of the target structure. It recursively splits the structure and thereby derives a binary decomposition tree rooted in *T *and whose leaves correspond to *T*-substructures. Each non-leaf node of this tree represents a substructure obtained by merging the two substructures of its respective children. Given this tree, RNA-SSD performs a stochastic local search, starting at the leaves, subsequently working its way up to the root.

**INFO-RNA **constructs sequences folding into a given secondary structure by employing a dynamic programming method for finding a well suited initial sequence. This sequence has a lowest energy with respect to the *T*. Since the latter does not necessarily fold into *T*, (due to potentially existing competing configurations) INFO-RNA then utilizes an improved (relative to the local search routine used in RNAinverse) stochastic local search in order to find a sequence in the neutral network of *T*. In contrast to RNAinverse, INFO-RNA allows for increasing the distance to the target structure. At the same time, only positions that do not pair correctly and positions adjacent to these are examined.

### 2.1 Cross

Cross is an *ab initio *folding algorithm that maps RNA sequences into 3-noncrossing RNA structures. It is guaranteed to search all 3-noncrossing, *σ*-canonical structures and derives some (not necessarily unique), loop-based mfe-configuration. In the following we always assume *σ *≥ 3. The input of Cross is an arbitrary RNA sequence *s *and an integer *N*. Its output is a list of *N *3-noncrossing, *σ*-canonical structures, the first of which being the mfe-structure for *s*. This list of *N *structures (*C*_0_, *C*_1_, ..., *C*_*N*-1_) is ordered by the free energy and the first list-element, the mfe-structure, is denoted by Cross(*s*). If no *N *is specified, Cross assumes *N *= 1 as default.

Cross generates a mfe-structure based on specific loop-types of 3-noncrossing RNA structures. For a given structure *S*, let *α *be an arc contained in *S *(*S*-arc) and denote the set of *S*-arcs that cross *α *by . For two arcs *α *= (*i, j*) and *α*' = (*i', j'*), we next specify the partial order "≺" over the set of arcs:

All notions of minimal or maximal elements are understood to be with respect to ≺. An arc *α *∈  is called a minimal, *β*-crossing if there exists no *α' *∈  such that *α' *≺ *α*. Note that *α *∈  can be minimal *β*-crossing, while *β *is not minimal *α*-crossing. 3-noncrossing diagrams exhibit the following four basic loop-types:

**(1) **A hairpin-loop is a pair

where (*i, j*) is an arc and [*i, j*] is an interval, i.e. a sequence of consecutive, isolated vertices (*i*, *i *+ 1, ..., *j *- 1, *j*).

**(2) **An interior-loop, is a sequence

where (*i*_2_*, j*_2_) is nested in (*i*_1_*, j*_1_). That is we have *i*_1 _*< i*_2 _*< j*_2 _*< j*_1_.

**Figure 11:**
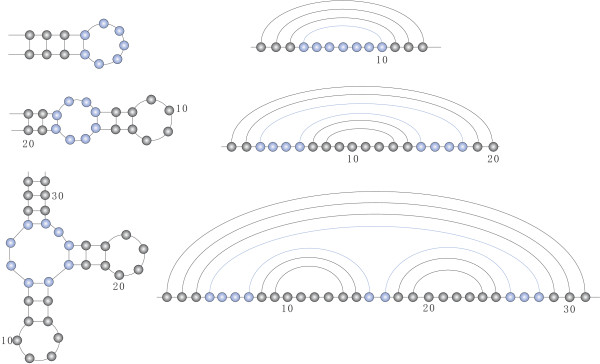
**The standard loop-types**. The standard loop-types: hairpin-loop (top), interior-loop (middle) and multi-loop (bottom). These represent all loop-types that occur in RNA secondary structures.

**(3) **A multi-loop, see Figure 11[19], is the closed structure formed by(3)

where  denotes the substructure over the interval [*ω*_*h*_, *τ*_*h*_], subject to the condition that if all these substructures are simply stems, then there are at least two of them, see Figure 6.

**Figure 12:**
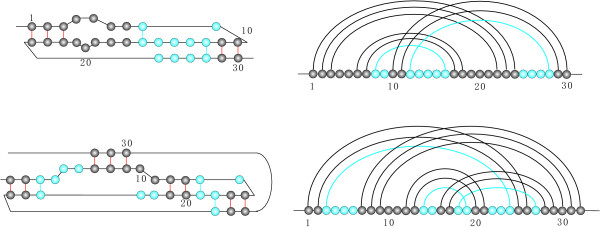
**Pseudoknots**. Pseudoknot loops, formed by all blue vertices and arcs.

A pseudoknot, see Figure 12[19], consists of the following data:

(P1) A set of arcs

where *i*_1 _= min{*i*_*h*_} and *j*_*t *_= max{*j*_*h*_}, such that

**(i) **the diagram induced by the arc-set *P *is irreducible, i.e. the dependency-graph of *P *(i.e. the graph having *P *as vertex set and in which *α *and *α' *are adjacent if and only if they cross) is connected and

**(ii) **for each (*i*_*h*_, *j*_*h*_) ∈ *P *there exists some arc *β *(not necessarily contained in *P*) such that (*i*_*h*_, *j*_*h*_) is minimal *β*-crossing.

(P2) Any *i*_1 _<*x *<*j*_*t*_, not contained in hairpin-, interior- or multi-loops.

Having discussed the basic loop-types, we are now in position to state

**Theorem 1 ***Any 3-noncrossing RNA pseudoknot structure has a unique loop-decomposition *[19].

**Figure 13:**
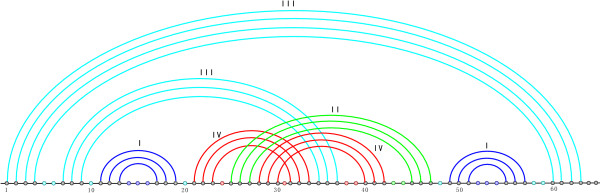
**Loop decomposition**. Here a hairpin-loop (I), an interior-loop (II), a multi-loop (III) and a pseudoknot (IV).

Figure 13 illustrates the loop decomposition of a 3-noncrossing structure.

In order to discuss the organization of Cross, we introduce the basic idea behind motifs and skeleta, combinatorial structures used in the folding algorithm.

**Figure 14:**
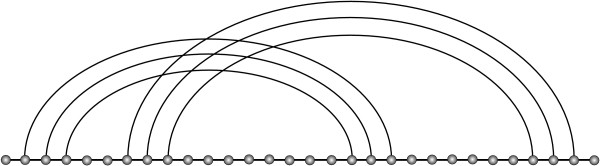
**Motif**. A 3-noncrossing, 3-canonical motif.

A motif is a 3-noncrossing structure, having only ≺-maximal stacks of size exactly *σ*, i.e. no stacks nested in other stacks, see Figure 14. Despite that motifs can exhibit complicated crossings, they can be inductively generated. A skeleton, *S *is a *k*-noncrossing structure such that

• its core, *c*(*S*) has no noncrossing arcs and

• its *L*-graph, *L*(*S*) is connected.

**Figure 15:**
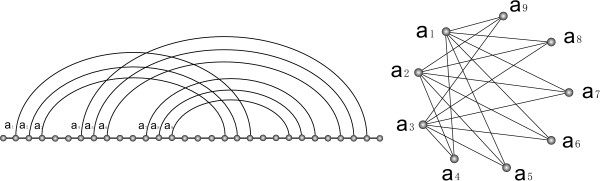
**Skeleton and its *L*-graph**. We display a skeleton (left) and its *L*-graph (right).

Here the core of a structure, *c*(*S*), is obtained by collapsing its stacks into single arcs (thereby reducing its length) and the graph *L*(*S*) is obtained by mapping arcs into vertices and connecting any two if they cross in the diagram representation of *S*, see Figure 15. A skeleton reflects all cross-serial interactions of a structure.

Having introduced motifs and skeleta we can proceed by discussing the general idea of Cross. The algorithm generates 3-noncrossing RNA structure "from top to bottom" via the following three subroutines:

**I **(SHADOW): In this routine we generate all maximal stacks of the structure. Note that a stack is maximal with respect to ≺ if it is not nested in some other stack. This is derived by "shadowing" the motifs, i.e. their *σ*-stacks are extended "from top to bottom".

**II **(SKELETONBRANCH): Given a shadow, the second step of Cross consists in generating, the skeleta-tree. The nodes of this tree are particular 3-noncrossing structures, obtained by successive insertions of stacks. Intuitively, a skeleton encapsulates all cross-serial arcs that cannot be recursively computed. Here the tree complexity is controlled via limiting the (total) number of pseudoknots.

**III **(SATURATION): In the third subroutine each skeleton is saturated via DP-routines. After the saturation the mfe-3-noncrossing structure is derived.

**Figure 16:**
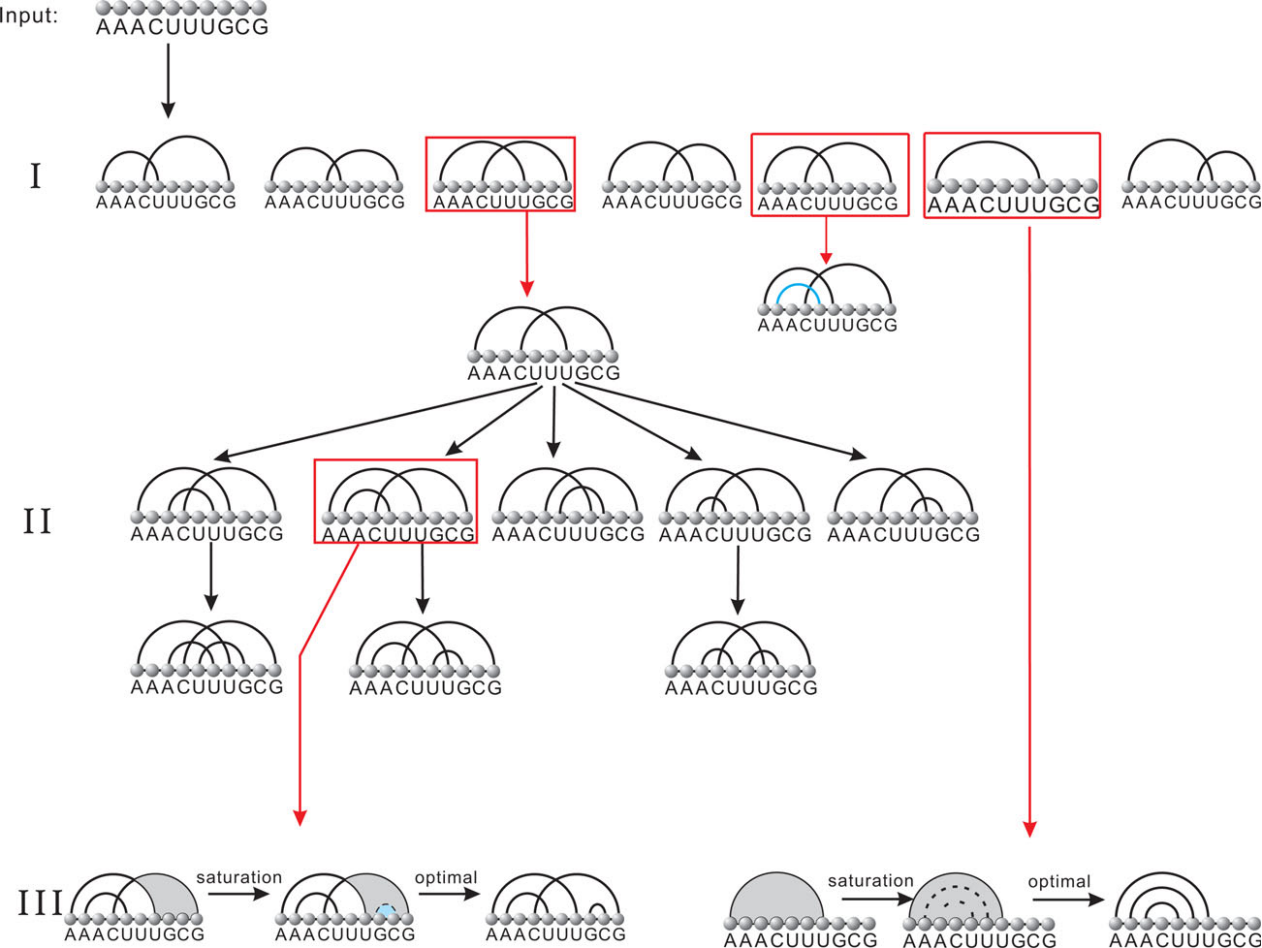
**An outline of **Cross. For illustration purposes we assume here *σ *= 1. The routines SHADOW, SKELETONBRANCH and SATURATION are depicted. Due to space limitations we only represent a few select motifs and for the same reason only one of the motifs displayed in the first row is extended by one arc (drawn in blue). Furthermore note that only motifs with crossings give rise to nontrivial skeleton-trees, all other motifs are considered directly as input for SATURATION.

Figure 16 provides an overview on how the three subroutines are combined.

## 3 The algorithm

The inverse folding algorithm Inv is based on the *ab initio *folding algorithm Cross. The input of Inv is the target structure, *T*. The latter is expressed as a character string of ":( )[ ]{ }", where ":" denotes unpaired base and "( )", "[ ]", "{ }" denote paired bases.

In Algorithm 7.1, we present the pseudocodes of algorithm Inv. After validation of the target structure (lines 2 to 5 in Algorithm 7.1), similar to INFO-RNA, Inv constructs an initial sequence and then proceeds by a stochastic local search based on the loop decomposition of the target. This sequence is derived via the routine ADJUST-SEQ. We then decompose the target structure into loops and endow these with a linear order. According to this order we use the routine LOCAL-SEARCH in order to find for each loop a "proper" local solution.

### 3.1 ADJUST-SEQ

In this section we describe Steps 2 and 3 of the pseudocodes presented in Algorithm 7.1. The routine MAKE-START, see line 8, generates a random sequence, *start*, which is compatible to the target, with uniform probability.

We then initialize the variable *seq*_min _via the sequence *start *and set the variable *d *= + ∞, where *d *denotes the structure distance between Cross(*seq*_min_) and *T*.

Given the sequence *start*, we construct a set of potential "competitors", *C*, i.e. a set of structures suited as folding targets for *start*. In Algorithm 7.2 we show how to adjust the start sequence using the routine ADJUST-SEQ. Lines 3 to 36 of Algorithm 7.2, contain a **For**-loop, executed at most  times. Here the loop-length  is heuristically determined.

For all computer experiments setting the Cross-parameter *N *= 50, the subroutine executed in the loop-body consists of the following three steps.

**Step I. Generating ***C*^0^(*λ*^*i*^) **via **Cross. Suppose we are in the *i*th step of the **For**-loop and are given the sequence *λ*^*i*-1 ^where *λ*^0 ^= *start*. We consider Cross(*λ*^*i*-1^, *N*), i.e. the list of suboptimal structures with respect to *λ*^*i*-1^,

If , then Inv returns *λ*^*i*-1^. Else, in case of , we set

Otherwise we do not update *seq*_min _and go directly to Step II.

**Step II. The competitors**. We introduce a specific procedure that "perturbs" arcs of a given RNA pseudoknot structure, *S*. Let *a *be an arc of *S *and let *l*(*a*), *r*(*a*) denote the start- and end-point of *a*. A perturbation of *a *is a procedure which generates a new arc *a'*, such that

**Figure 17:**
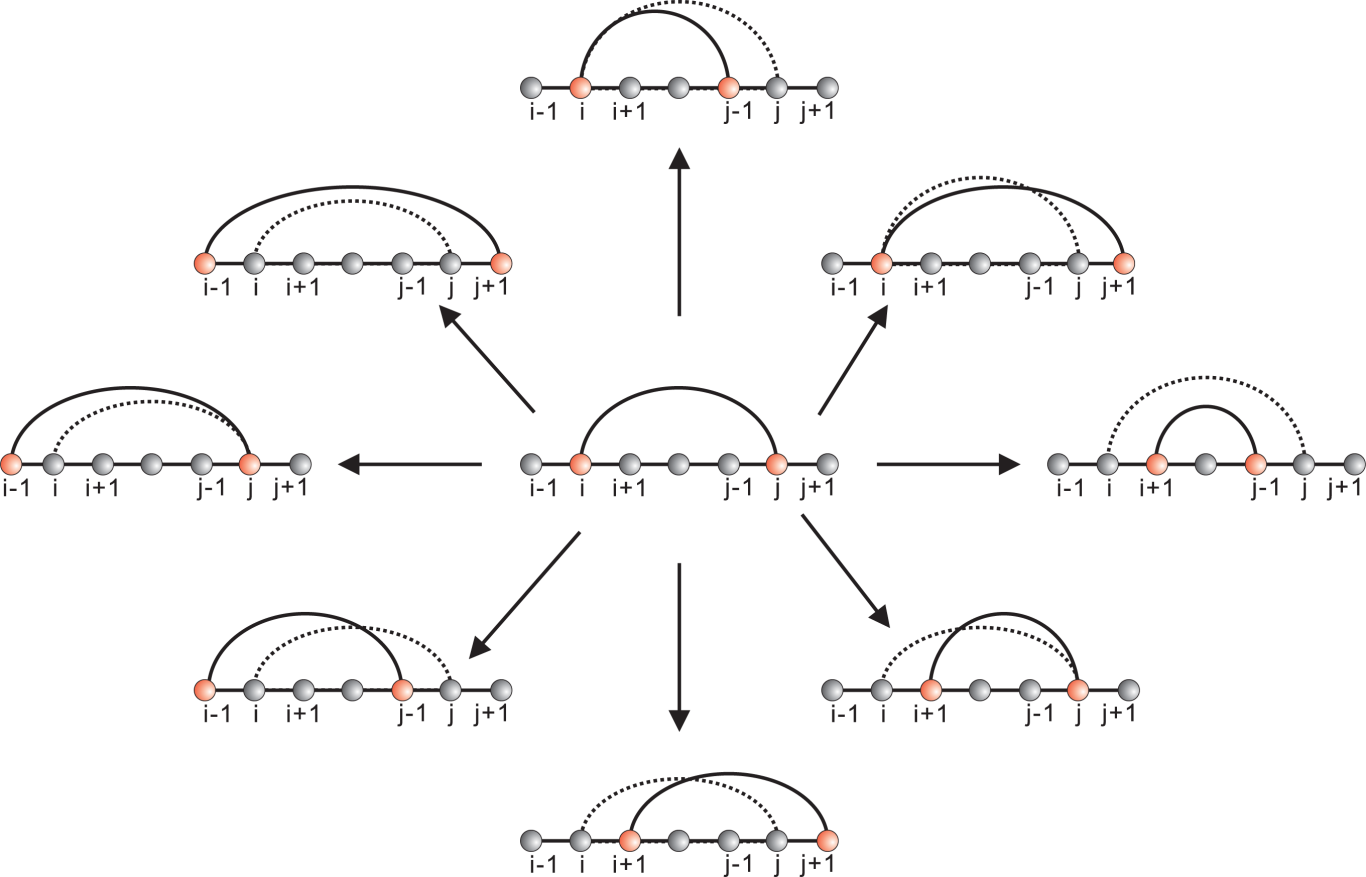
**Perturbations**. Nine perturbations of an arc (*i, j*). Original arcs are drawn dotted, and the arcs incident to red bases are the perturbations.

Clearly, there are nine perturbations of any given arc *a *(including *a *itself), see Figure 17.

We proceed by keeping *a*, replacing the arc *a *by a nontrivial perturbation or remove *a*, arriving at a set of ten structures *ν*(*S, a*).

Now we use this method in order to generate the set *C*^1^(*λ*^*i*-1^) by perturbing each arc of each structure . If  has *A*_*h *_arcs, , then

**Figure 18:**
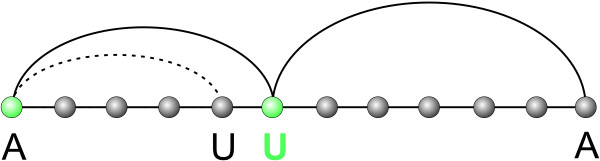
**Inconsistent structures**. The dotted arc is perturbed by shifting its end-point. This perturbation leads to a nucleotide establishing two base pairs, which is impossible.

**Figure 19:**
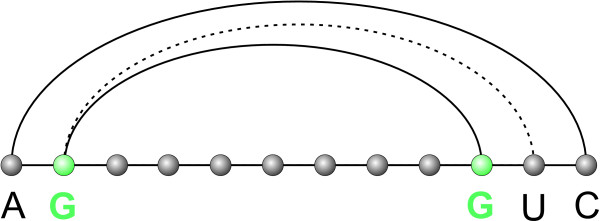
**Incompatible structures**. We display a perturbation of the dotted arc leading to a structure that is incompatible to the given sequence.

**Figure 20:**
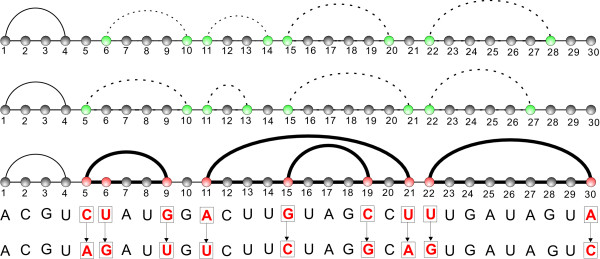
**Sequence mutation**. Suppose the top and middle structures represent the set of competitors and the bottom structure is target. We display *λ*^*i *- 1^(top sequence) and its mutation, *λ*^*i *^(bottom sequence). Two nucleotides of base pairs not contained in *T *are colored green, nucleotides subject to mutations are colored red.

**Figure 21:**
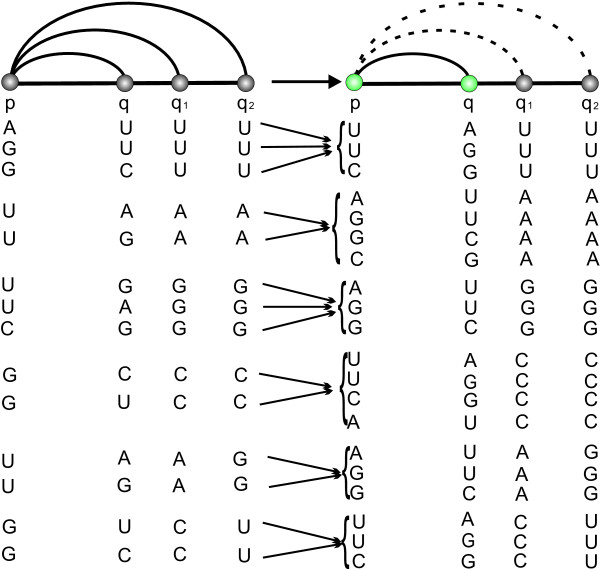
**Sequence adjust**. Mutations are always possible, suppose *p *is paired with *q *in *T *and *p *is paired with *q*_1 _in one competitor and *q*_2 _in another one. For a fixed nucleotide at *p *there are at most two scenarios, since a base can pair with at most two different bases. For instance, for **G **we have the pairs **G-C,G-U**. We display all nucleotide configurations (LHS) and their corresponding solutions (RHS).

This construction may result in duplicate, inconsistent or incompatible structures. Here, a structure is inconsistent if there exists at least one position paired with more than one base, and incompatible if there exists at least one arc not compatible with *λ*^*i*-1^, see Figures 18 and 19. Here compatibility is understood with respect to the Watson-Crick and **G**-**U **base pairing rules. Deleting inconsistent and incompatible structures, as well as those identical to the target, we arrive at the set of competitors,

**Step III. Mutation**. Here we adjust *λ*^*i*-1 ^with respect to *T *as well as the set of competitors, *C*(*λ*^*i*-1^) derived in the previous step. Suppose . Let *p*(*S*, *w*) be the position paired to the position *w *in the RNA structure *S *∈ *C*(*λ*^*i*-1^), or 0 if position *w *is unpaired. For instance, in Figure 20, we have *p*(*T*, 1) = 4, *p*(*T*, 2) = 0 and *p*(*T*, 4) = 1. For each position *w *of the target *T*, if there exists a structure *C*_*h*_(*λ*^*i*-1^) ∈ *C*(*λ*^*i*-1^) such that *p*(*C*_*h*_(*λ*^*i*-1^, *w*) ≠ *p*(*T*, *w*) (see positions 5, 6, 9, and 11 in Figure 20) we modify *λ*^*i*-1 ^as follows:

1. **unpaired position**: If *p*(*T, w*) = 0, we update  randomly into the nucleotide , such that for each *C*_*h*_(*λ*^*i*-1^) ∈ *C*(*λ*^*i*-1^), either *p*(*C*_*h*_(*λ*^*i*-1^), *w*) = 0 or  is not compatible with  where *v *= *p*(*C*_*h*_(*λ*^*i*-1^), *w*) < 0, See position 6 in Figure 20.

2. **start-point**: If *p*(*T*, *w*) <*w*, set *v *= *p*(*T*, *w*), We randomly choose a compatible base pair () different from (, ) such that for each *C*_*h*_(*λ*^*i*-1^) ∈ *C*(*λ*^*i*-1^), either *p*(*C*_*h*_(*λ*^*i*-1^), *w*) = 0 or  is not compatible with , where *u *= *p*(*C*_*h*_(*λ*^*i*-1^), *w*) > 0 is the end-point paired with  in *C*_*h*_(*λ*^*i*-1^) (Figure 20: (5, 9). The pair **G-C **retains the compatibility to (5, 9), but is incompatible to (5, 10)). By Figure 21 we show feasibility of this step.

3. **end-point**: If 0 <*p*(*T*, *w*) <*w*, then by construction the nucleotide has already been considered in the previous step.

Therefore, updating all the nucleotides of *λ*^*i*-1^, we arrive at the new sequence .

Note that the above mutation steps heuristically decrease the structure distance. However, the resulting sequence is not necessarily incompatible to all competitors. For instance, consider a competitor *C*_*h *_whose arcs are all contained *T*. Since *λ*^*i *^is compatible with *T*, *λ*^*i *^is compatible with *C*_*h*_. Since competitors are obtained from suboptimal folds such a scenario may arise.

In practice, this situation represents not a problem, since these type of competitors are likely to be ruled out by virtue of the fact that they have a mfe larger than that of the target structure.

Accordingly we have the following situation, competitors are eliminated due to two, equally important criteria: incompatibility as well as minimum free energy considerations.

If the distance of Cross(*λ*^*i*^) to *T *is less than or equal to *d*_min _+ 5, we return to Step I (with *λ*^*i*^). Otherwise, we repeat Step III (for at most 5 times) thereby generating  and set  where *d*(Cross(), *T*) is minimal.

The procedure ADJUST-SEQ employs the negative paradigm [16] in order to exclude energetically close conformations. It returns the sequence *seq*_middle _which is tailored to realize the target structure as mfe-fold.

### 3.2 DECOMPOSE and LOCAL-SEARCH

In this section we introduce two the routines, DECOMPOSE and LOCAL-SEARCH. The routine DECOMPOSE partitions *T *into linearly ordered energy independent components, see Figure 13 and Section 2.1. LOCAL-SEARCH constructs iteratively an optimal sequence for *T *via local solutions, that are optimal to certain substructures of *T*.

DECOMPOSE: Suppose *T *is decomposed as follows,

where the *T*_*w *_are the loops together with all arcs in the associated stems of the target.

We define a linear order over *B *as follows: *T*_*w *_<*T*_*h *_if either

1. *T*_*w *_is nested in *T*_*h*_, or

2. the start-point of *T*_*w *_precedes that of *T*_*h*_.

**Figure 22:**
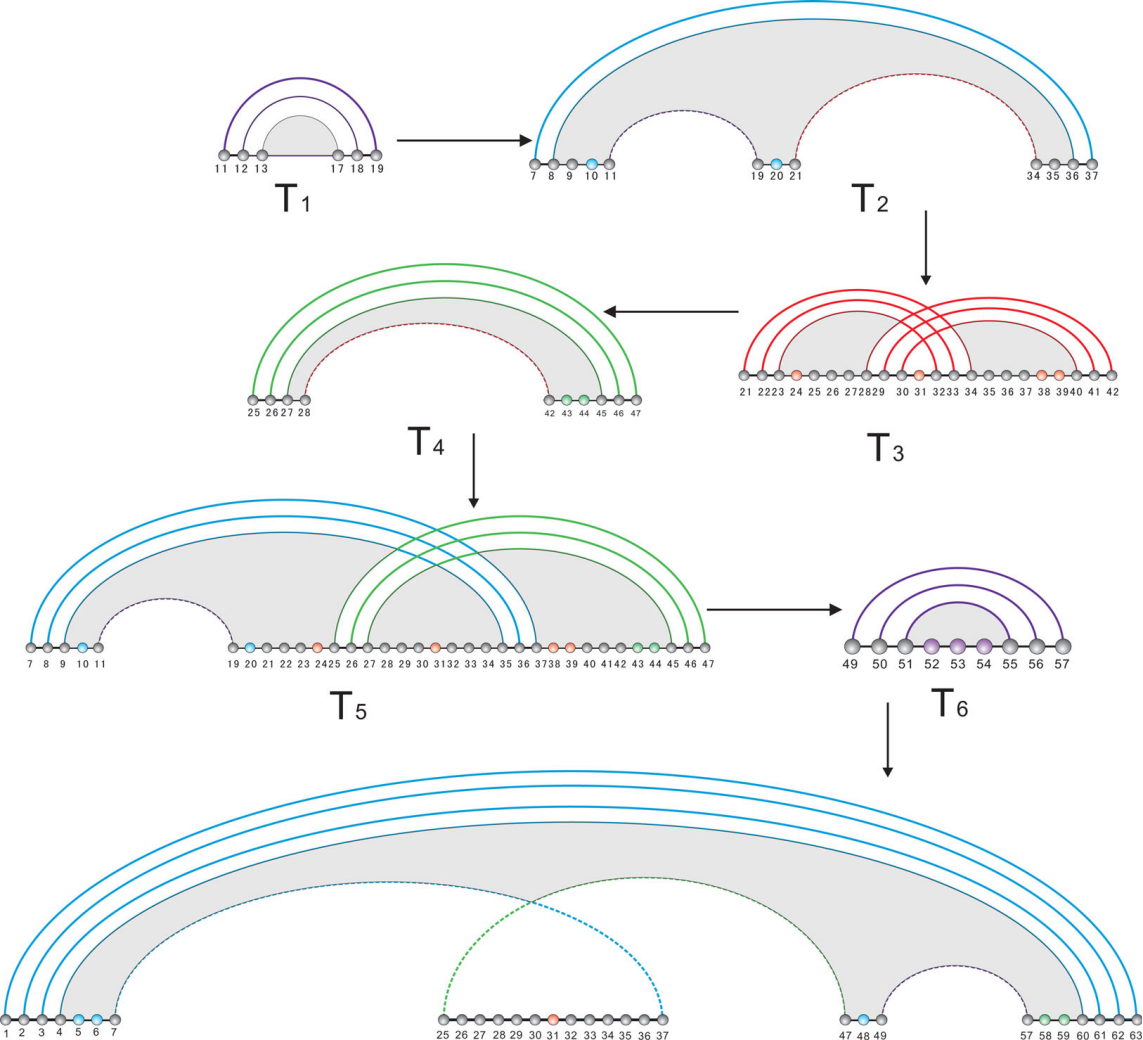
**Ordering**. Linear ordering of loops: *a*_1 _= [11, 19], *b*_1 _= [10, 20], *a*_2 _= [7, 37], *b*_2 _= [5, 39], *a*_3 _= [21, 42], *b*_3 _= [20, 44], *a*_4 _= [25, 47], *b*_4 _= [24, 48], *a*_5 _= [7, 47], *b*_5 _= [5, 48], *a*_6 _= [49, 57], *b*_6 _= [48, 59], *a*_7 _= [1, 63], *b*_7 _= [1, 65].

In Figure 22 we display the linear order of the loops of the structure shown in Figure 13.

Next we define the interval

projecting the loop *T*_*w *_onto the interval [*l*(*T*_*w*_), *r*(*T*_*w*_)] and *b*_*w *_= [*l'*, *r'*] ⊃ *a*_*w*_, being the maximal interval consisting of *a*_*w *_and its adjacent unpaired consecutive nucleotides, see Figure 13. Given two consecutive loops *T*_*w *_<*T*_*w *+ 1_, we have two scenarios:

• either *b*_*w *_and *b*_*w*+1 _are adjacent, see *b*_5 _and *b*_6 _in Figure 22,

• or *b*_*w *_⊆ *b*_*w *+ 1_, see *b*_1 _and *b*_2 _in Figure 22.

**Figure 23:**
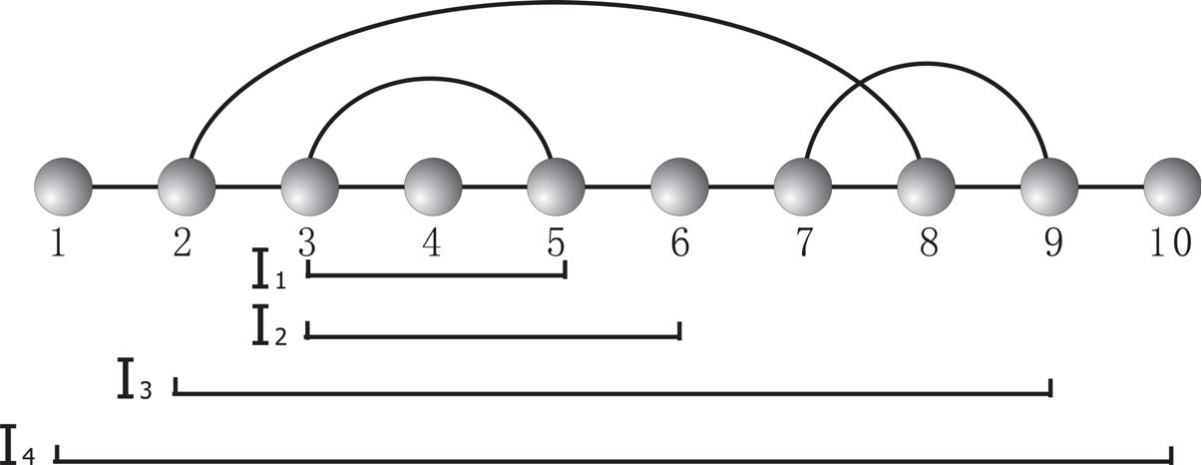
**Example of the interval sequence**. Loops and their induced sequence of intervals.

Let , then we have the sequence of intervals *a*_1_, *b*_1_, *c*_1_, ..., *a*_*m'*_, *b*_*m'*_, *c*_*m'*_. If there are no unpaired nucleotides adjacent to *a*_*w*_, then *a*_*w *_= *b*_*w *_and we simply delete all such *b*_*w*_. Thereby we derive the sequence of intervals *I*_1_, *I*_2_, ..., *I*_*m*_. In Figure 23 we illustrate how to obtain this interval sequence: here the target decomposes into the loops *T*_1_, *T*_2 _and we have *I*_1 _= [3, 5], *I*_2 _= [3, 6], *I*_3 _= [2, 9], and *I*_4 _= [1, 10].

LOCAL-SEARCH: Given the sequence of intervals *I*_1_, *I*_2_, ...,  *I*_*m*_. We proceed by performing a local stochastic search on the subsequences  (initialized via *seq *= *seq*_middle _and where *s*|_[*x*, *y*] _= *s*_*x*_*s*_*x *+ 1 _... *s*_*y*_). When we perform the local search on , only positions that contribute to the distance to the target, see Figure 10, or positions adjacent to the latter, will be altered. We use the arrays *U*_1_, *U*_2 _to store the unpaired and paired positions of *T*. In this process, we allow for mutations that increase the structure distance by five with probability 0.1. The latter parameter is heuristically determined. We iterate this routine until the distance is either zero or some halting criterion is met.

## 4 Discussion

The main result of this paper is the presentation of the algorithm Inv, freely available at http://www.combinatorics.cn/cbpc/inv.html

**Figure 24:**
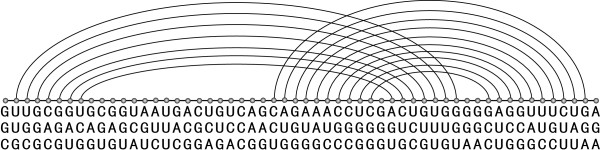
**The UTR pseudoknot of bovine coronavirus**. Its diagram representation and several sequences of its neutral network as constructed by Inv.

Its input is a 3-noncrossing RNA structure *T*, given in terms of its base pairs (*i*_1_, *i*_2_) (where *i*_1 _<*i*_2_). The output of Inv is an RNA sequences *s *= (*s*_1_*s*_2_...*s*_*n*_), where *s*_*h *_∈ {**A, C, G, G**} with the property Cross(*s*) = *T*, see Figure 24.

The core of Inv is a stochastic local search routine which is based on the fact that each 3-noncrossing RNA structure has a unique loop-decomposition, see Theorem 1 in Section 2.1. Inv generates "optimal" subsequences and eventually arrives at a global solution for *T *itself. Inv generalizes the existing inverse folding algorithm by considering arbitrary 3-noncrossing canonical pseudoknot structures. Conceptually, Inv differs from INFO-RNA in how the start sequence is being generated and the particulars of the local search itself.

**Figure 25:**
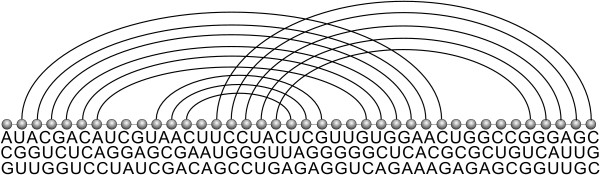
**Pseudoknot PKI**. The Pseudoknot PKI of the internal ribosomal entry site (IRES) region [41], its diagram representation and three sequences of its neutral network as constructed by Inv.

As discussed in the introduction it has to be given an argument as to *why *the inverse folding of pseudoknot RNA structures works. While folding maps into RNA secondary structures are well understood, the generalization to 3-noncrossing RNA structures is nontrivial. However the combinatorics of RNA pseudoknot structures [27,28,38] implies the existence of large neutral networks, i.e. networks composed by sequences that all fold into a specific pseudoknot structure. Therefore, the fact that it is indeed possible to generate via Inv sequences contained in the neutral networks of targets against competing pseudoknot configurations, see Figure 24 and Figure 25 confirms the predictions of [31].

**Figure 26:**
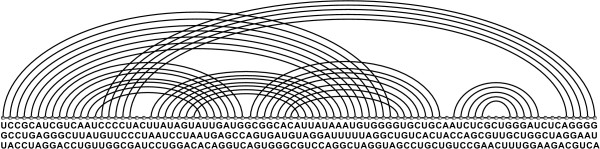
**Example of nonplanar structure**. A nonplanar 3-noncrossing RNA structure together with three sequences realizing them as mfe-structures.

An interesting class are the 3-noncrossing nonplanar pseudoknot structures. A nonplanar pseudoknot structure is a 3-noncrossing structure which is not a bi-secondary structure in the sense of Stadler [30]. That is, it cannot be represented by noncrossing arcs using the upper and lower half planes. Since DP-folding paradigms of pseudoknots folding are based on gap-matrices [15], the minimal class of "missed" structures (given the implemented truncations) are exactly these, nonplanar, 3-noncrossing structures. In Figure 26 we showcase a nonplanar RNA pseudoknot structure and 3 sequences of its neutral network, generated by Inv.

As for the complexity of Inv, the determining factor is the subroutine LOCAL-SEARCH. Suppose that the target is decomposed into *m *intervals with the length ℓ_1_, ...., ℓ_*m*_. For each interval, we may assume that line 2 of LOCAL-SEARCH runs for *f*_*h *_times, and that line 14 is executed for *g*_*h *_times. Since LOCAL-SEARCH will stop (line 4) if *T*_*start *_= *T *(line 3), the remainder of LOCAL-SEARCH, i.e. lines 7 to 41 run for (*f*_*h *_- 1) times, each such execution having complexity O(ℓ_*h*_). Therefore we arrive at the complexity

where c(ℓ) denotes the complexity of the Cross. The multiplicities *f*_*h *_and *g*_*h *_depend on various factors, such as *start*, the random order of the elements of *U*_1_, *U*_2 _(see Algorithm 7.3) and the probability *p*. According to [32] the complexity of c(ℓ_*h*_) is  and accordingly the complexity of Inv is given by

**Figure 27:**
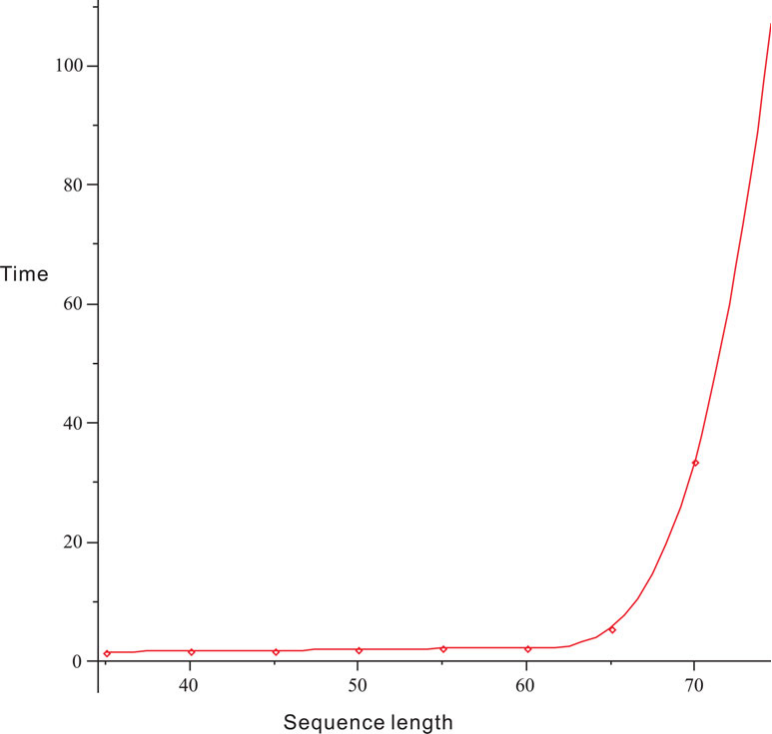
**Fitting of mean inverse folding time (seconds) over sequence length via 2 cubic spines**. For *n *= 35, ..., 75 we choose a natural pseudoknot structure from the PKdatabase and display the average inverse folding time based on sampling 200 sequences of the neutral network of the respective target.

In Figure 27 we present the average inverse folding time of several natural RNA structures taken from the PKdatabase [39]. These averages are computed via generating 200 sequences of the target's neutral networks. In addition we present in Table 1 the total time for 100 executions of Inv for an additional set of RNA pseudoknot structures.

**Table 1:** Inverse folding times for 100 executions of Inv for various RNA pseudoknot structures.

RNA structure	length	trials	total time	success rate
TPK-70.28 [42]	40	100	4 m 57.81s	100%

Ec_PK2 [43]	59	100	5 m 33.28s	100%
PMWaV-2 [44]	62	100	1 m 7.12s	100%

tRNA	76	100	5 m 2.49s	100%

In all cases all trials generated successfully sequences of the respective neutral networks.

## 5 Competing interests

The authors declare that they have no competing interests.

## 6 Authors' contributions

All authors contributed equally to this paper. All authors read and approved the final manuscript.

## 7 Appendix

**7.1 Algorithm 7.1 **- INVERSE-FOLD

**Input**: *k*-noncrossing target structure *T*

**Output**: an RNA sequence *seq*

**Require**: *k *≤ 3 and *T *is composed of ":( ) [ ] { }"

**Ensure**: Cross(*seq*) = *T*

1. ▻ Step 1: Validate structure

2. **if false **= CHECK-STRU(*T*) **then**

3.    **print **incorrect structure

4.    **return **NIL

5. **end if**

6.

7. ▻ Step 2: Generate the start sequence

8. *start *← MAKE-START(*T*)

9.

10. ▻ Step 3: Adjust the start sequence

11. *seq*_middle _← ADJUST-SEQ(*start, T*)

12.

13. ▻ Step 4: Decompose *T *and derive the ordered intervals.

14. Interval array *I*

15. *m *← |*I*| ▻ *I *satisfies *I*_*m *_= *T*

16.

17. ▻ Step 5: Stochastic Local Search

18. *seq *← *seq*_middle_

19. **for all **intervals in the array *I*_*w *_**do**

20.    *l *← start-point(*I*_*w*_)

21.    *r *← end-point(*I*_*w*_)

22.    *s' *← *seq*|_[*l*, *r*] _▻ get sub-sequence

23.    *seq*|_[*l*, *r*] _LOCAL-SEARCH(*s'*, *I*_*w*_)

24. **end for**

25.

26. ▻ Step 6: output

27. **if ***seq*_min _= Cross(*seq*) **then**

28.    **return ***seq*

29. **else**

30.    **print **Failed!

31.    **return **NIL

32. **end if**

**7.2 Algorithm 7.2 **- ADJUST-SEQ

**Input**: the original start sequence *start*

**Input**: the target structure *T*

**Output**: an initialized sequence *seq*_middle_

1. *n *← length of *T*

2. *d*_min _← + ∞, *seq*_min _← *start*

3. **for ***i *= 1 to  do

4.    ▻ Step I: generate the set *C*^0^(*λ*^*i *- 1^) via Cross

5.    *C*^0^(*λ*^*i *- 1^) ← Cross(*λ*^*i *- 1^, *N*)

6.    

7. **if ***d *= 0 **then**

8.       **return ***λ*^*i *- 1^

9.    **else if ***d *<*d*_min _then

10.       *d*_min _← *d*, *seq*_min _← *λ*^*i *- 1^

11.    **end if**

12.

13.    ▻ Step II: generate the competitor set *C*(*λ*^*i *- 1^)

14.    *C*^1^(*λ*^*i*-1^) ← *ϕ*

15.    **for all ** ∈ *C*^1^(*λ*^*i*-1^) **do**

16.       **for all **arc  of **do**

17.          

18.       **end for**

19.    **end for**

20.    *C*(*λ*^*i *- 1^) =

21.    { is valid}

22.

23.    ▻ Step III: mutation

24.    *seq *← *λ*^*i *- 1^

25.    **for ***w *= 1 to *n ***do**

26.       **if **∃ *C*_*h*_(*λ*^*i*-1^) ∈ *C*(*λ*^*i*-1^) s.t. *p*(*C*_*h*_, *w*) ≠ *p*(*T*, *w*) **then**

27.          *seq*[*w*] ← random nucleotide or pair, s.t. *seq *∈ *C*[*T*] and *seq *∉ *C*[*C*_*h*_(*λ*^*i*-1^)]

28.       **end if**

29.    **end for**

30.    *T*_*seq *_← Cross(*seq*)

31.    **if ***d*(*T*_*seq*_, *T*) <*d*_min _+ 5 **then**

32.       *seq*_middle _← *seq*

33.    **else if **Step III run less than 5 times **then**

34.       **goto **Step III

35.    **end if**

36. **end for ▻ **loop to line 3

37.

38. **return ***seq*_middle_

**7.3 Algorithm 7.3 **- LOCAL-SEARCH

**Input:***seq*_middle_

**Input**: the target *T*

Output: *seq*

**Ensure**: Cross(*seq*) = *T*

1. *seq *← *seq*_middle_

2. **if **Cross(*seq*) = *T ***then**

3.    return *seq*

4. **end if**

5. decompose *T *and derive the ordered intervals

6. *I *← [*I*_1_, *I*_2_, ..., *I*_*m*_]

7. for all *I_w _*in *I *do

8.    ▻ Phase I: Identify positions

9.     ▻ initialize *d*_min_

10.

11.    derive *U*_1 _via 

12.    derive *U*_2 _via 

13.

14.    ▻ Phase II: Test and Update

15.    **for all ***p *in *U*_1 _**do**

16.       random *T *compatible mutate *seq*_*p*_

17.    **end for**

18.    **for all **[*p, q*] in *U*_2 _**do**

19.       random *T *compatible mutate *seq*_*p*_

20.    **end for**

21.

22.    *E *← *ϕ*

23.    **for all ***p *∈ *U*_1_, *U*_2 _**do**

24.       *d *← *d*(*T*, Cross(*seq*_*p*_))

25.       **if ***d *<*d*_*min *_**then**

26.          *d*_min _← *d*, *seq *← *seq*_*p*_

27.          **goto **Phase I

28.       **else if ***d*_*min *_<*d *<*d*_*min *_+ 5 **then**

29.          **goto **Phase I with the probability 0.1

30.       **end if**

31.       **if ***d *= *d*_*min *_**then**

32.          *E *← *E *∪ {*seq*}

33.       **end if**

34.    **end for**

35.    *seq *← *e*_0 _∈ *E*, where *e*_0 _has the lowest mfe in *E*

36.    **if **Phase I run less than 10 *n *times **then**

37.       **goto **Phase I

38.    **end if**

39. **end for**

40. **return ***seq*

## 8 Acknowledgements

We are grateful to Fenix W.D. Huang for discussions. Special thanks belongs to the two anonymous referee's whose thoughtful comments have greatly helped in deriving an improved version of the paper. This work was supported by the 973 Project, the PCSIRT of the Ministry of Education, the Ministry of Science and Technology, and the National Science Foundation of China.
